# Supplementary figures and images for: Coordinated action of multiple transporters in the acquisition of essential cationic amino acids by the intracellular parasite Toxoplasma gondii
Source: PLoS Pathog. 2021 Aug 25;17(8):e1009835. doi: 10.1371/journal.ppat.1009835 (PMC8423306; doi:10.1371/journal.ppat.1009835)

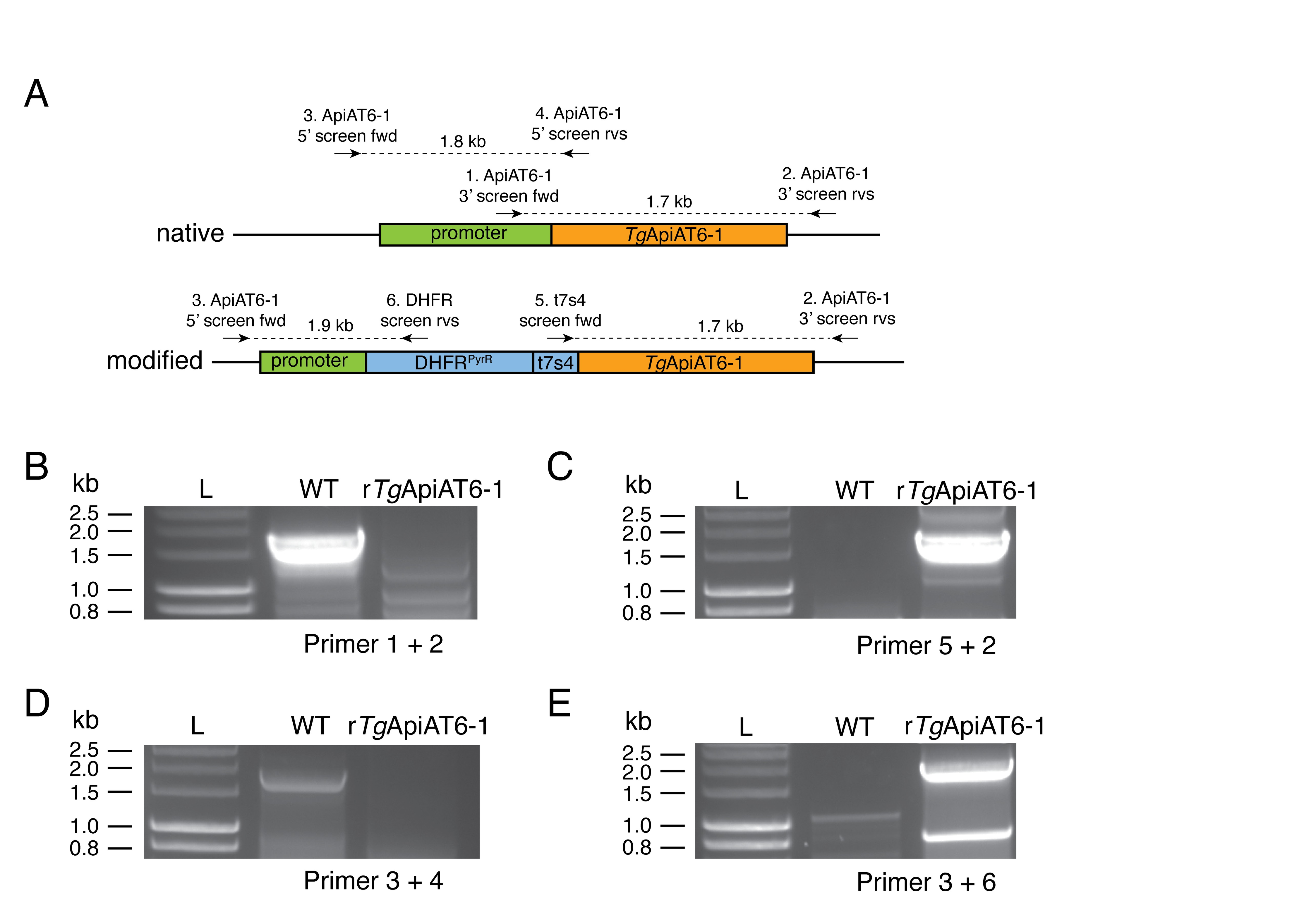

Supplement: S1 Fig — A. Schematic depicting the promoter replacement strategy to generate the ATc-regulated TgApiAT6-1 strain (rTgApiAT6-1), and the positions of screening primers used in subsequent experiments to validate successful promoter replacement. The native locus (top) and promoter-replaced locus (bottom) are shown. DHFRPyrR, pyrimethamine-resistant dihydrofolate reductase cassette; t7s4, ATc-regulatable teto7-sag4 promoter. The teto7-sag4 promoter is bound by a tetracycline-controlled transactivator protein that facilitates transcription of the downstream gene (TgApiAT6-1 in this instance). The addition of the tetracycline analogue ATc to the culture medium results in binding of ATc to the transactivator protein, which inhibits binding of the transactivator protein to the teto7-sag4 promoter, and consequently reduces transcription of the downstream gene [71]. B-E. PCR analysis using genomic DNA extracted from native RH strain (WT) and modified rTgApiAT6-1 strain parasites, with primers that specifically detect the 3’ region of the native locus (B), the 3’ region of the modified locus (C), the 5’ region of the native locus (D), and the 5’ region of the modified locus (E). (TIF) [file ppat.1009835.s001.tif]

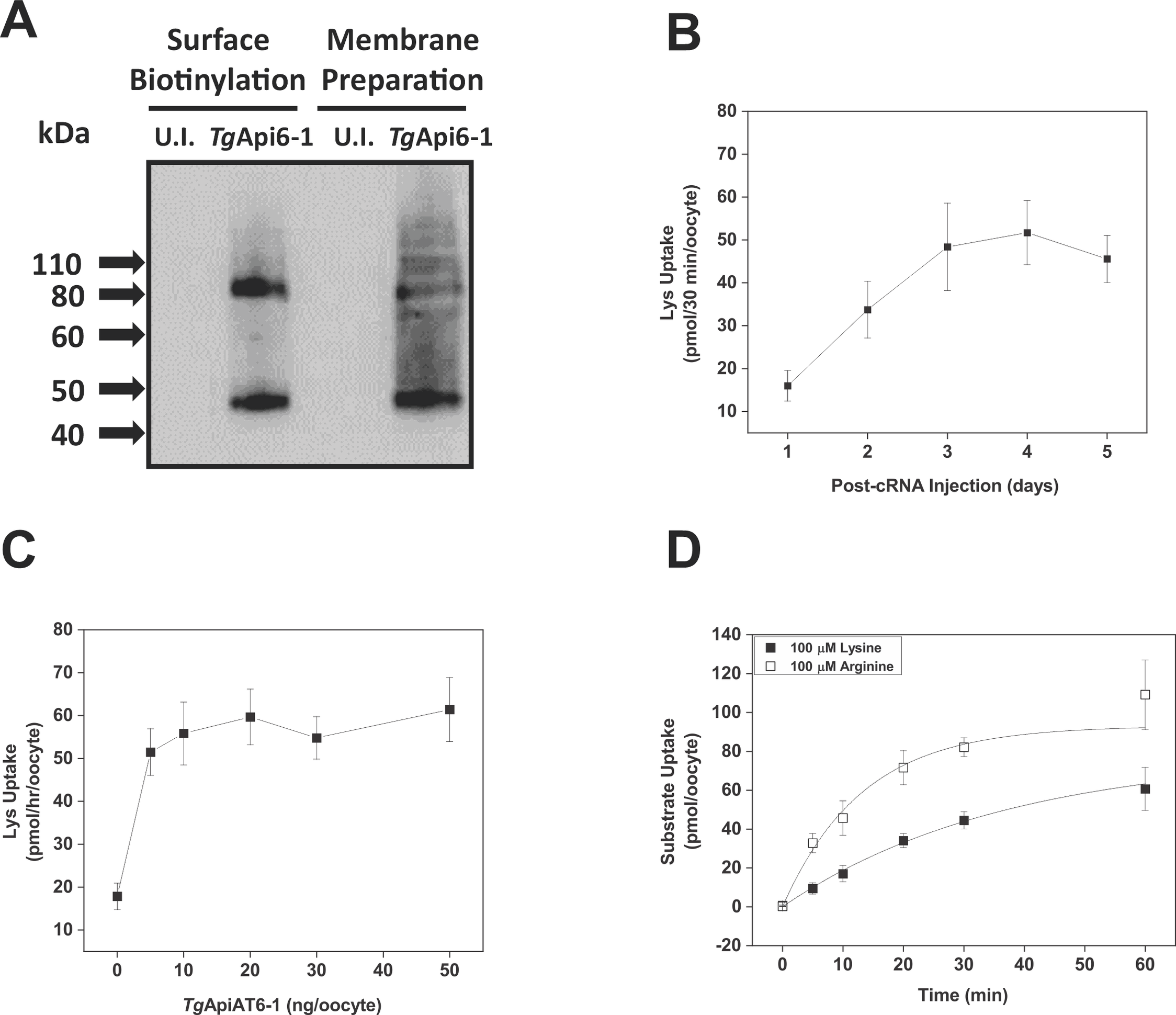

Supplement: S2 Fig — A. Western blot with anti-HA antibodies to detect proteins from surface-biotinylated and total membrane fractions of oocytes injected with TgApiAT6-1 cRNA or uninjected (U.I.) controls. Each lane contains protein equivalents from equal oocyte numbers. B. Time-course measuring Lys uptake in TgApiAT6-1-expressing in oocytes one to five days post-cRNA injection. Each data point represents the mean ± S.D. uptake of 10 oocytes for a single experiment, and is representative of three independent experiments. Uptake was measured in the presence of 100 μM unlabelled Lys and 1.0 μCi/ml [14C]Lys. The uptake of Lys in uninjected oocytes has been subtracted for all days post-cRNA injection tested. C. Lys uptake in TgApiAT6-1-expressing in oocytes injected with 0–50 ng of TgApiAT6-1-encoding cRNA. Each data point represents the mean ± S.D. uptake of 10 oocytes for a single experiment and are representative of three independent experiments. Uptake was measured in the presence of 100 μM unlabelled Lys and 1.0 μCi/ml [14C]Lys. All measurements were conducted on day 4 post-cRNA injection. The uptake of Lys in a single uninjected oocyte batch has been subtracted from all data points. D. Time-course of Lys (open squares) and Arg (closed squares) uptake into TgApiAT6-1 expressing oocytes for determination of initial rate conditions. Uptake was measured in the presence of 100 μM unlabelled Arg and 1.0 μCi/ml [14C]Arg or 100 μM unlabelled Lys and 1.0 μCi/ml [14C]Lys. Each data point represents the mean ± S.D. uptake of 10 oocytes for a single experiment, and is representative of three independent experiments. The uptake of Lys or Arg at the same concentration in uninjected oocytes was subtracted from all data points. (TIF) [file ppat.1009835.s002.tif]

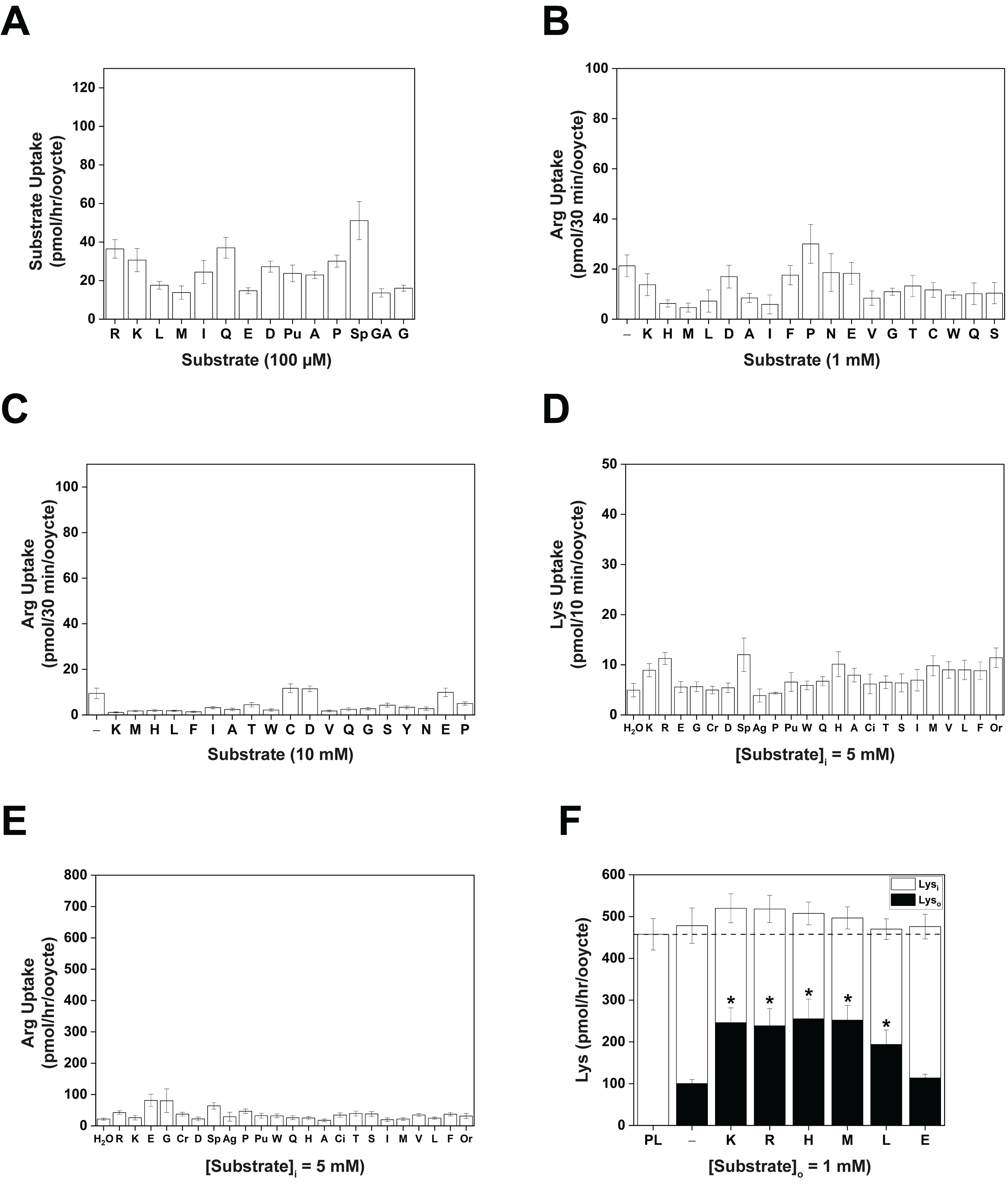

Supplement: S3 Fig — A. Uptake of a range of amino acids into oocytes not expressing TgApiAT6-1. Uptake was measured in the presence of 100 μM unlabelled substrate and 1.0 μCi/ml [3H] or [14C] substrate. Amino acid substrates are represented by single letter codes, while for other metabolites: Pu, putrescine; Sp, spermidine; and GA, γ-amino butyric acid (GABA). Each bar represents the mean ± S.D. uptake of 10 oocytes for a single experiment paired with TgApiAT6-1 expressing oocytes from Fig 2B, and each is representative of three independent experiments. B-C. Uptake of 100 μM unlabelled Arg and 1.0 μCi/ml [14C]Arg was measured in oocytes not expressing TgApiAT6-1 in the presence of 1 mM (B) or 10 mM (C) of the competing amino acid. Amino acid substrates are represented by single letter codes. Each bar represents the mean ± S.D. uptake of 10 oocytes for a single experiment paired with TgApiAT6-1 expressing oocytes from Fig 2C and 2D, and are representative of three independent experiments. The first bar in each graph represents the Arg-only uptake control. D-E. Oocytes not expressing TgApiAT6-1 (injected with H2O) were pre-loaded by microinjecting the indicated substrates to a final concentration of ~5 mM and the uptake of 15 μM Lys and 1.0 μCi/ml of [14C]Lys (D) or 1 mM Arg and 1.0 μCi/ml of [14C]Arg (E) was measured. Amino acid substrates are represented by single letter codes, while for other metabolites: Cr, creatine; Ag, agmatine; Sp, spermidine; Pu, putrescine; Ci, citrulline; and Or, ornithine. Each bar represents the mean ± S.D. uptake of 10 oocytes for a single experiment paired with TgApiAT6-1 expressing oocytes from Fig 5E and 5F, and are representative of three independent experiments. F. Lys efflux and retention in TgApiAT6-1 expressing oocytes in the presence of candidate trans-stimulating substrates. TgApiAT6-1-injected oocytes were pre-loaded over 8 hrs with 1 mM unlabelled Lys and 1.0 μCi/ml of [14C]Lys (TgApiAT6-1). Efflux of pre-loaded Lys (Lyso; black bars) and retent [file ppat.1009835.s003.tif]

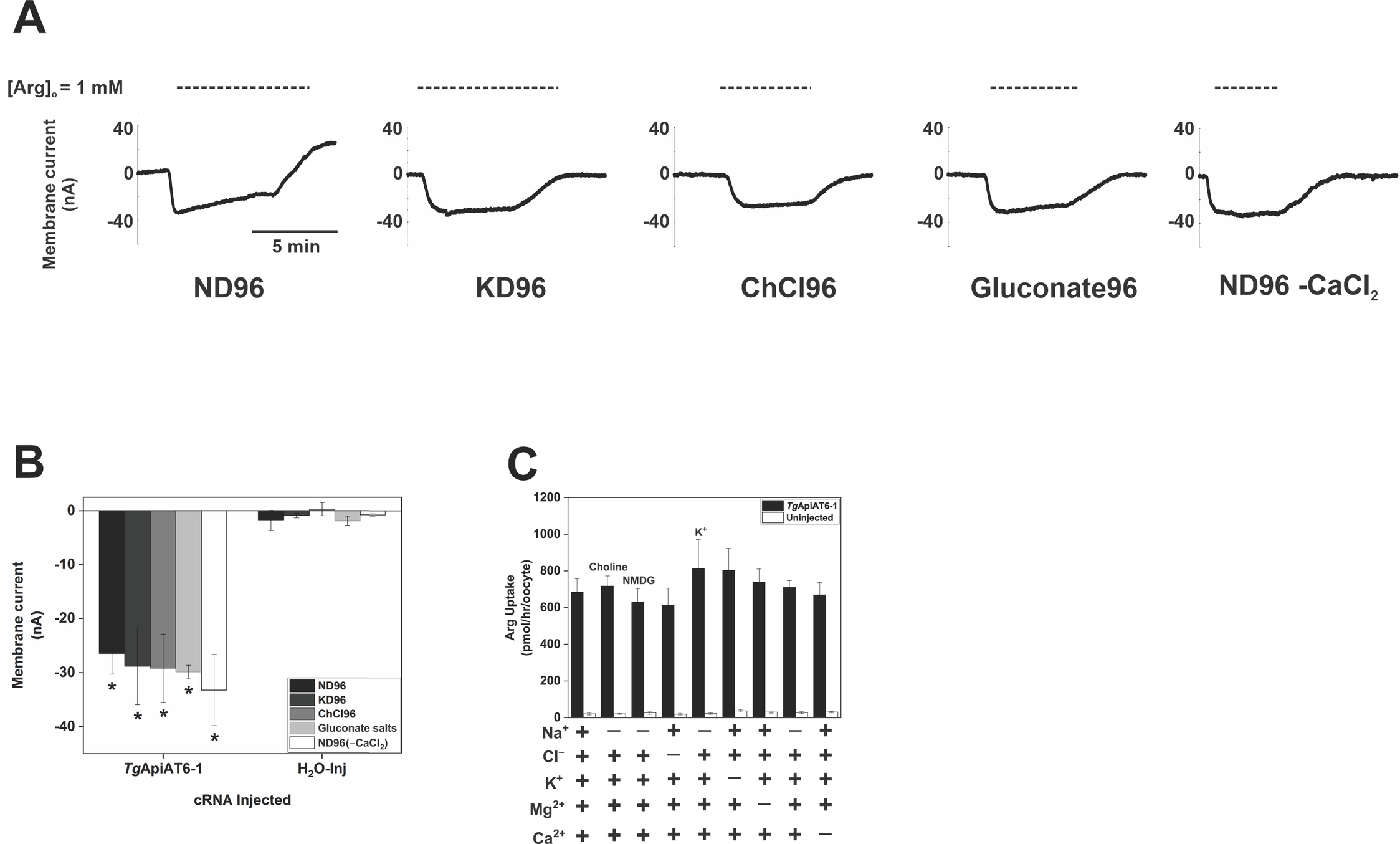

Supplement: S4 Fig — A. Paired representative membrane current recordings recorded from TgApiAT6-1 expressing oocytes superfused with 1 mM Arg in the presence of different extracellular salt compositions. The membrane current recordings were made under voltage-clamp configuration (Em = −50mV). Superfusion buffers indicated below tracings are: ND96 (Na+ buffer), KD96 (Na+ replaced by K+), Gluconate (Cl- replaced by gluconate), ChCl96 (Na+ replaced by choline), ND96 −CaCl2 (Ca2+ removed). All recordings were made at pH 7.3. Full buffer compositions are listed in S3 Table. B. Membrane current recordings in TgApiAT6-1 expressing oocytes as described in A for voltage-clamp recordings. Each bar represents the mean ± S.D. of inward currents from 8–12 oocytes per condition. Statistical analysis compares the mean of TgApiAT6-1 expressing oocytes with their buffer pair in H2O-injected oocytes (*, P < 0.05, one-way ANOVA, Dunnett’s post-hoc test). Note that all currents in A-B were recorded in two-voltage clamp configuration set to a membrane potential of −50 mV to record membrane current or in unclamped mode for recording membrane potential. C. The uptake of Arg in TgApiAT6-1 expressing oocytes incubated in different buffer compositions. Uptake was measured in the presence of 1 mM unlabelled Arg and 1.0 μCi/ml [14C]Arg. The removed salt from standard ND96 buffer (pH 7.3) is indicated below the graph. Na+-replacement salts are indicated above their respective bars. Each bar represents the mean ± S.D. uptake of 10 oocytes for a single experiment, and are representative of three independent experiments. Statistical analysis compares all bars of TgApiAT6-1 expressing oocytes incubated in different buffer compositions (P > 0.05; one-way ANOVA, Dunnett’s post-hoc test). (TIF) [file ppat.1009835.s004.tif]

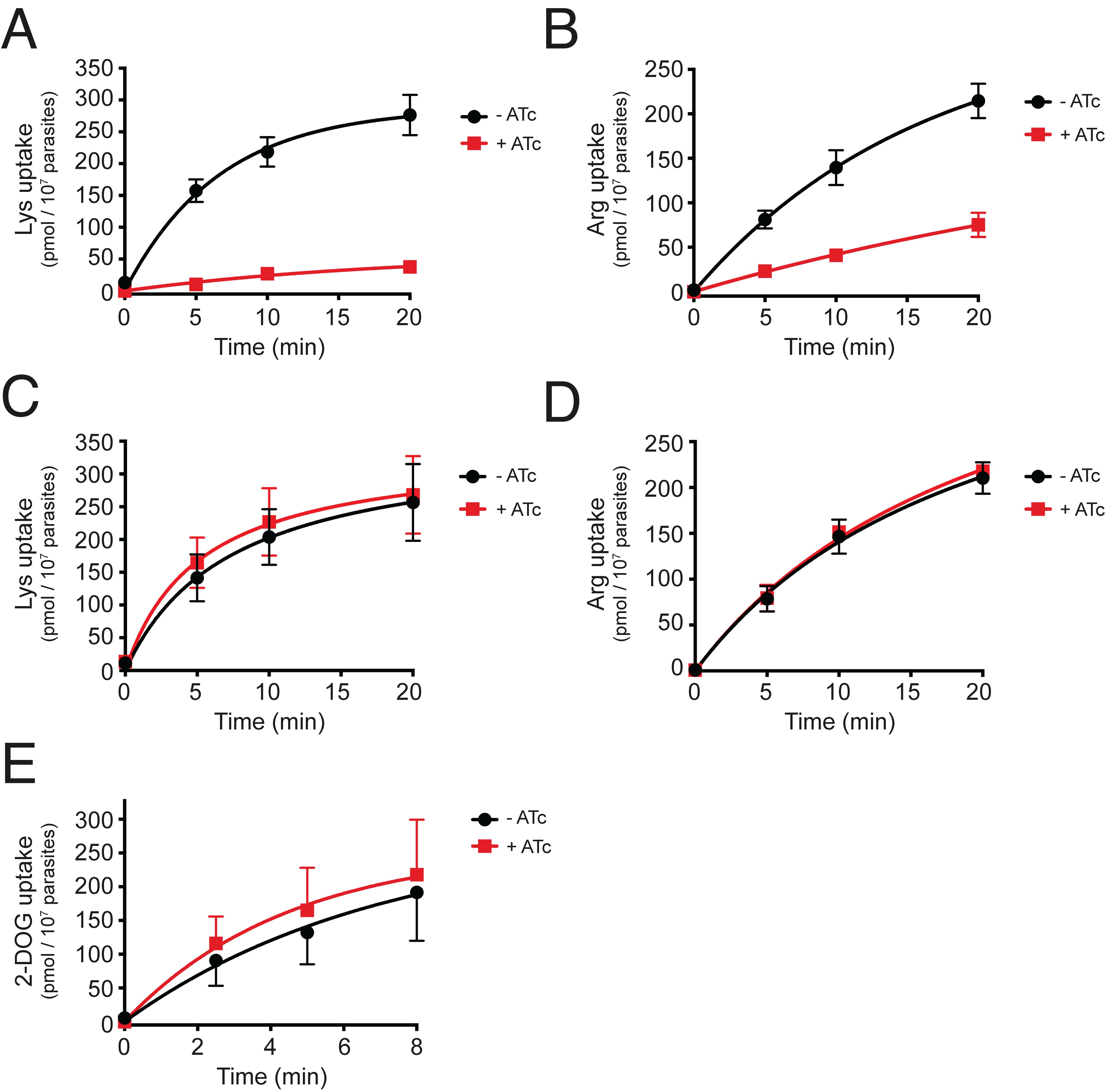

Supplement: S5 Fig — A-D. Uptake of Lys (A, C) or Arg (B, D) in rTgApiAT6-1 (A, B) or WT (C, D) parasites across a 20 min time-course. Lys uptake was measured in a solution containing 50 μM unlabelled Lys and 0.1 μCi/ml [14C]Lys. Arg uptake was measured in a solution containing 80 μM unlabelled Arg and 0.1 μCi/ml [14C]Arg. E. Uptake of 2-deoxyglucose (2-DOG) in rTgApiAT6-1 parasites across an 8 min time-course. 2-DOG uptake was measured in 25 μM unlabelled 2-DOG and 0.2 μCi/ml [14C]2-DOG. All data points represent the mean ± S.D. from three independent experiments. One phase exponential curves were fitted to the data. (TIF) [file ppat.1009835.s005.tif]

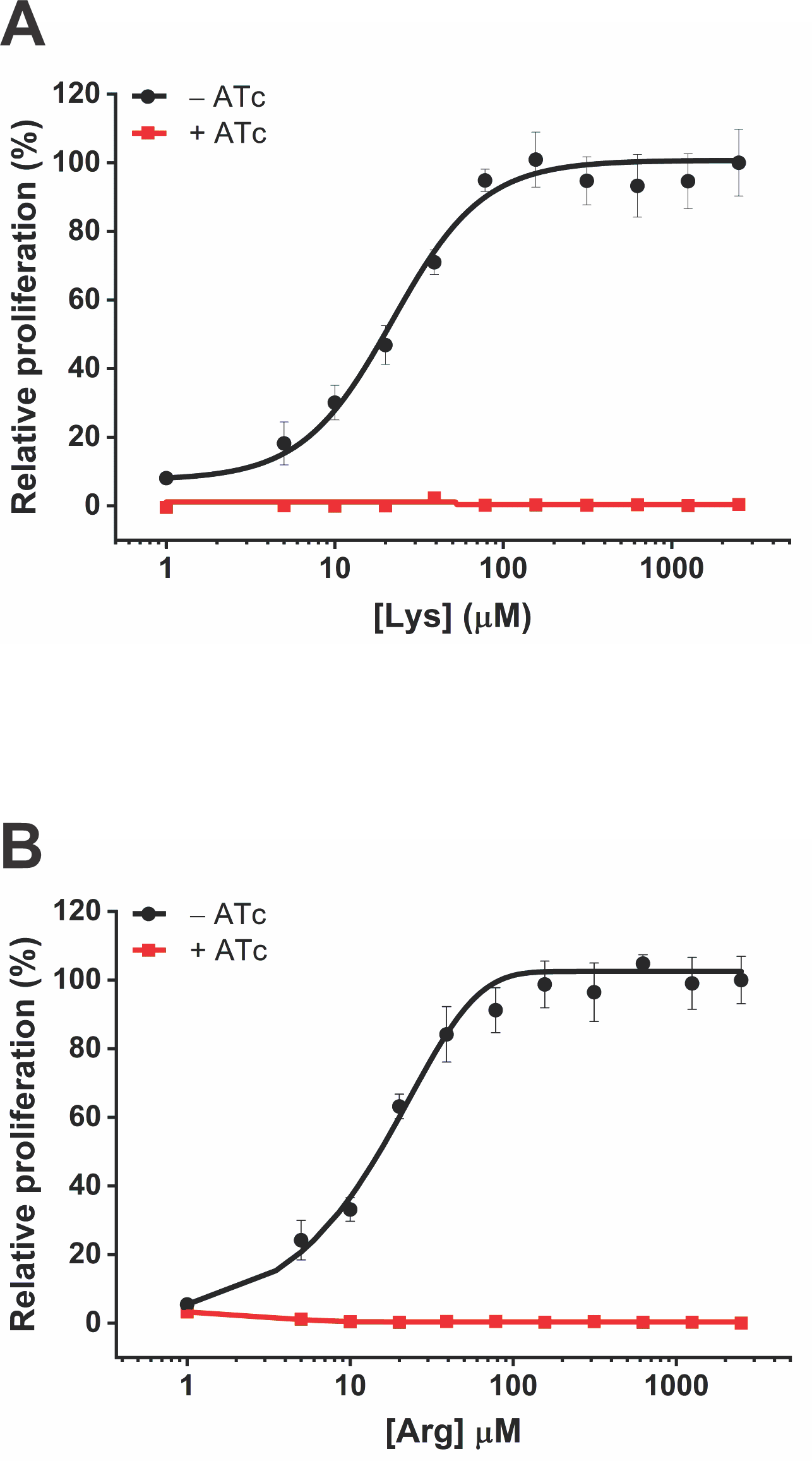

Supplement: S6 Fig — Fluorescence growth assays measuring proliferation of rTgApiAT6-1/Tomato parasites in either the absence (black) or presence (red) of ATc. Parasites were cultured in RPMI medium containing a range of [Lys] (1–2500 μM) with [Arg] constant at 200 μM (A) or a range of [Arg] (1–2500 μM) with [Lys] constant at 200 μM (B). Parasite proliferation at each amino acid concentration was expressed as a percentage of proliferation of parasites cultured at the highest Lys or Arg concentration when these parasites were at mid-log stage growth. Data points represent the mean ± S.D. of three independent experiments, each consisting of three technical replicates. (TIF) [file ppat.1009835.s006.tif]

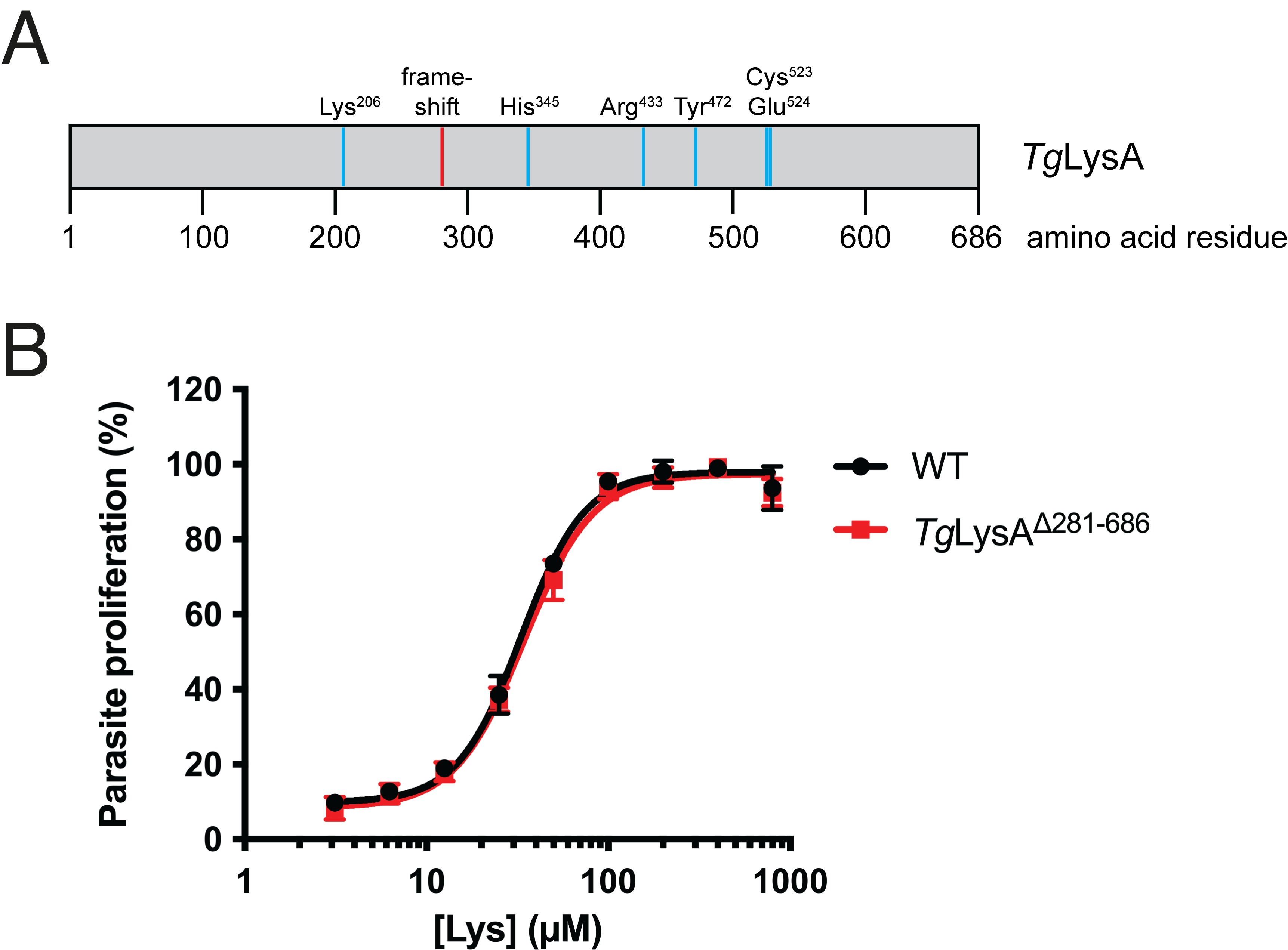

Supplement: S7 Fig — A. Scale diagram of the amino acid sequence of the TgLysA protein, with the positions of predicted active site residues depicted in cyan, and the position of the frameshift mutation generated in the lysAΔ281–68 strain depicted in red. B. Fluorescence growth assays measuring proliferation of WT (RH/Tomato; black) and lysAΔ281–68 (red) parasites cultured in RPMI medium containing a range of [Lys] (3–800 μM). Parasite proliferation is expressed as a percentage of growth in mid-log stage parasites cultured in 400 μM Lys. Data points represent the mean ± S.D. of four independent experiments, each consisting of three technical replicates. (TIF) [file ppat.1009835.s007.tif]

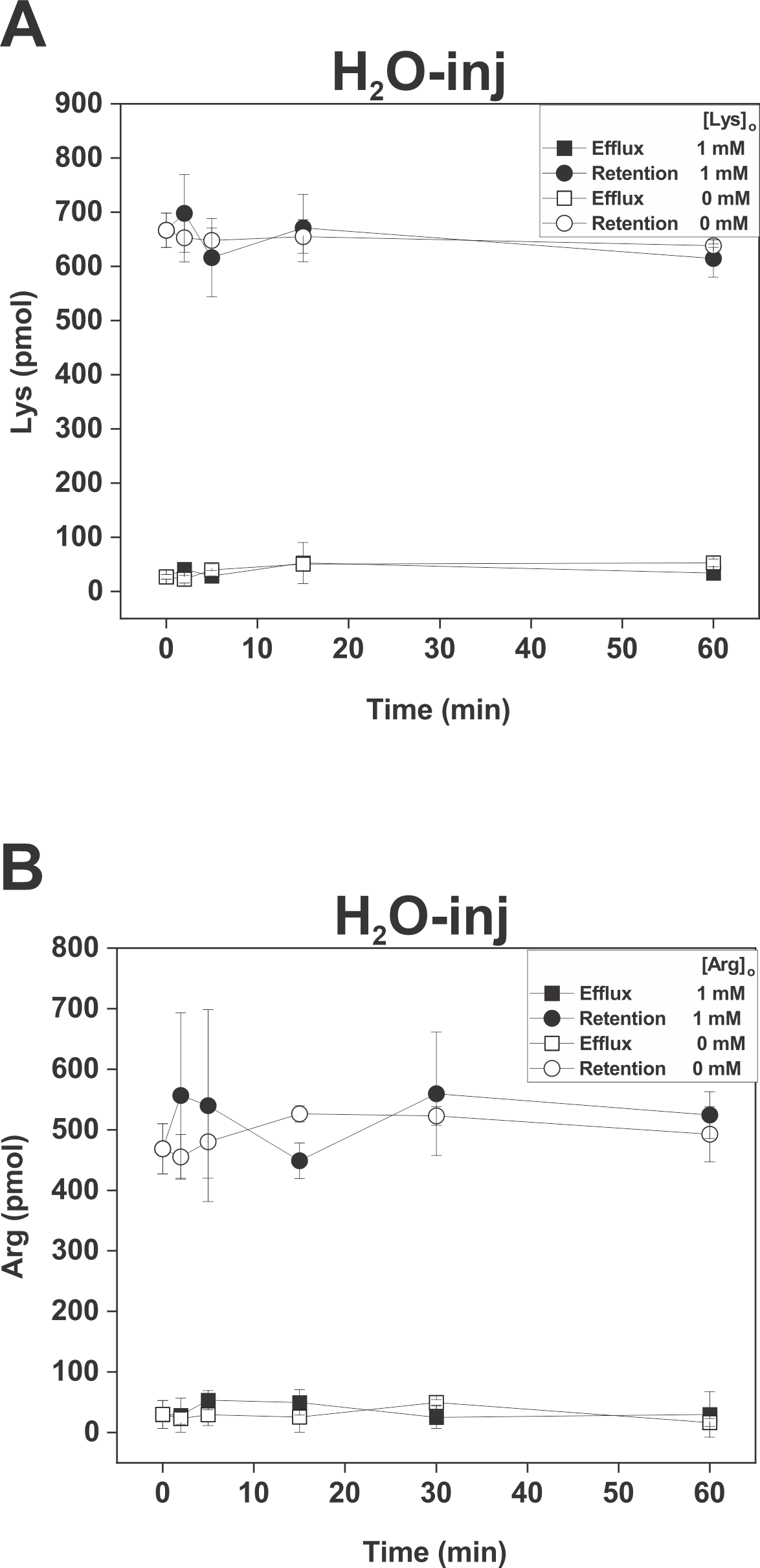

Supplement: S8 Fig — H2O-injected oocytes were pre-loaded with either 1 mM unlabelled Lys and 1.0 μCi/ml of [14C]Lys (A) or 1 mM unlabelled Arg and 1.0 μCi/ml of [14C]Arg (B) until they had reach the same approximate intracellular concentration of radiolabelled substrate (21–28 hr) as TgApiAT6-1- and TgApiAT1-expressing oocytes (Fig 5A and 5B). The retention of substrates was measured in the presence of 1 mM external substrate (closed symbols) or in the absence of an external substrate (open symbols). Data points represent the mean ± S.D. from 3 batches of 5 oocytes from one experiment, and are representative of 3 independent experiments. (TIF) [file ppat.1009835.s008.tif]

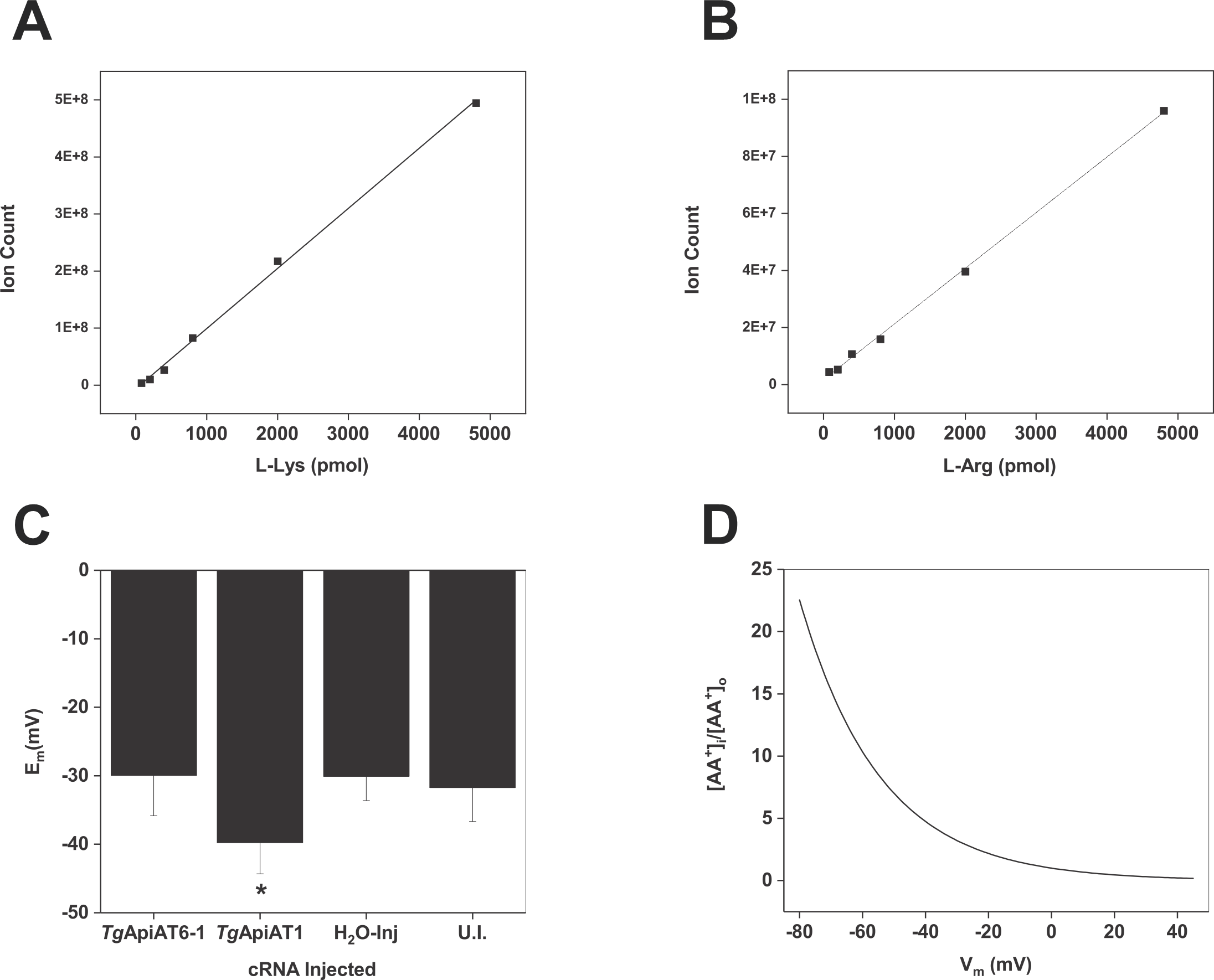

Supplement: S9 Fig — A-B. LC-MS/MS calibration curves for Lys (A) and Arg (B). The linear regressions were fitted with an R2 = 0.99 for both curves. C. The oocyte membrane potential (Em) of TgApiAT6-1 expressing oocytes, TgApiAT1 expressing oocytes, H2O-injected (H2O-inj) control oocytes and uninjected (U.I.) control oocytes were measured in unclamped mode. Resting Em measurements were conducted following incubation for 4 day post-cRNA injection in oocyte Ringer (OR2+) buffer, with oocytes transferred to ND96 buffer for recording. Oocytes with Em < −23 mV were discarded from further analysis. Each bar represents the mean ± S.D. of inward currents with the number of oocytes recorded as follows: TgApiAT6-1 (n = 16), TgApiAT1 (n = 19), H2O-injected (n = 13), and uninjected controls (n = 19). Statistical analysis compares all bars to uninjected controls (* P < 0.05, one-way ANOVA, Dunnett’s post-hoc test). D. The theoretical equilibrium distribution ([AA+]inside/[AA+]outside) vs membrane potential (Em) for a monovalent cation across a freely diffusible membrane as calculated by the Nernst equation (see Materials and Methods, Eq 2). (TIF) [file ppat.1009835.s009.tif]
