# Supplementary material for: Coordinated action of multiple transporters in the acquisition of essential cationic amino acids by the intracellular parasite Toxoplasma gondii
Source: PLoS Pathog. 2021 Aug 25;17(8):e1009835. doi: 10.1371/journal.ppat.1009835 (PMC8423306; doi:10.1371/journal.ppat.1009835)
Supplement: S1 Table — Included are the average retention time (R.T.) of the metabolite, the average mass-to-charge (m/z) ratio of the ion, the name of the metabolite, and the quality control standard deviation (QC Relative S.D). The fold change of the substrate and other detected metabolites was determined by dividing the value at 25hrs with the 0 hr value for TgApAT6-1 expressing and uninjected (U.I.) oocytes. (DOCX) [file ppat.1009835.s010.docx]

**S1 Table. Metabolite fold-change upon incubation of *Tg*ApiAT6-1-expressing oocytes in a solution containing 1 mM Lys for 25 hr.** Included are the average retention time (R.T.) of the metabolite, the average mass-to-charge (m/z) ratio of the ion, the name of the metabolite, and the quality control standard deviation (QC Relative S.D). The fold change of the substrate and other detected metabolites was determined by dividing the value at 25hrs with the 0 hr value for *Tg*ApAT6-1 expressing and uninjected (U.I.) oocytes.

| **Average R.T. (min)** | **Average m/z ion** | **Metabolite name** | ***Tg*ApiAT6-1**  **Fold change** | **U.I.**  **Fold change** | **QC Relative S.D.^†^ (%)** |
| --- | --- | --- | --- | --- | --- |
| 5.23 | 102.055 | 1-Aminocyclopropane-1-carboxylate | 1.28526 | 0.81708 | 5.81665 |
| 2.16 | 134.0728 | 2-Aminobenzimidazole | 1.11515 | 0.80961 | 3.77822 |
| 2.99 | 268.1031 | 2'-Deoxyguanosine | 1.47185 | 1.12645 | 23.33689 |
| 4.44 | 136.0754 | 2-Phenylacetamide | 1.13431 | 0.74061 | 15.83807 |
| 8.02 | 170.092 | 3-Methylhistidine | 1.4043 | 1.3079 | 14.2883 |
| 3.8 | 126.0665 | 5-Methylcytosine | 0.99075 | 0.90912 | 7.94482 |
| 2.51 | 204.1224 | Acetylcarnitine | 1.11484 | 0.84375 | 4.331 |
| 1.82 | 146.1173 | Acetylcholine | 1.46295 | 1.1988 | 14.19191 |
| 5.78 | 348.0688 | Adenosine 3'-monophosphate | 0.96334 | 0.62512 | 7.8574 |
| 5.19 | 90.0552 | Alanine | 0.92696 | 0.66869 | 3.13868 |
| 5.29 | 162.0757 | α-Aminoadipate | 17.18478 | 6.9136 | 9.02607 |
| 4.64 | 132.1016 | Aminocaproic acid | 1.11877 | 0.82564 | 7.62816 |
| 5.55 | 133.0605 | Asparagine | 1.5907 | 0.80082 | 2.23249 |
| 6.2 | 134.0444 | Aspartic acid | 1.42756 | 0.9783 | 3.79935 |
| 1.36 | 245.0942 | Biotin | 1.0781 | 1.03326 | 24.15659 |
| 1.78 | 232.1536 | Butyryl carnitine | 0.85838 | 0.52864 | 10.88052 |
| 3.14 | 104.107 | Choline | 1.456 | 0.87259 | 2.30584 |
| 5.81 | 176.1026 | Citrulline | 0.87012 | 0.90672 | 9.87419 |
| 5.04 | 132.0765 | Creatine | 1.17408 | 1.06766 | 2.23729 |
| 3.65 | 114.0662 | Creatinine | 1.00765 | 0.58729 | 8.14055 |
| 4.61 | 122.0269 | Cysteine | 1.25099 | 0.66153 | 7.8297 |
| 3.6 | 112.0506 | Cytosine | 2.421 | 1.74297 | 19.53398 |
| 3.04 | 146.1172 | Deoxycarnitine | 1.00606 | 0.93715 | 4.72925 |
| 8.46 | 130.0861 | D/L-Pipecolinic acid | 11.37571 | 2.31442 | 3.98697 |
| 1.73 | 220.1172 | D-Pantothenic acid | 0.97535 | 0.95606 | 18.78292 |
| 5.73 | 148.0601 | Glutamic acid | 0.83766 | 0.65862 | 6.40573 |
| 5.45 | 147.0761 | Glutamine | 3.50459 | 1.12665 | 3.77099 |
| 2.35 | 137.0455 | Hypoxanthine | 1.0818 | 0.83262 | 5.84812 |
| 2.93 | 269.0873 | Inosine | 0.80693 | 0.69797 | 23.27755 |
| 3.38 | 132.1017 | Isoleucine | 0.61492 | 0.44892 | 20.81187 |
| 1.75 | 104.0707 | L-3-Aminoisobutyric acid | 1.52624 | 1.02609 | 16.88848 |
| 5.73 | 130.0497 | L-5-Oxoproline | 0.80494 | 0.64069 | 5.87214 |
| 8.58 | 175.1186 | Arginine | 1.44454 | 0.83491 | 8.42061 |
| 4.04 | 177.039 | l-ascorbic acid | 1.30228 | 0.62337 | 28.56301 |
| 3.87 | 162.112 | L-Carnatine | 1.02329 | 0.90112 | 8.44223 |
| 3.15 | 132.1016 | Leucine | 0.27475 | 0.44136 | 13.67144 |
| 3.63 | 150.058 | Methionine | 1.04314 | 0.76224 | 13.16419 |
| 8.83 | 133.0969 | L-ornithine | 1.93007 | 1.09574 | 6.51922 |
| 8.46 | 147.1125 | Lysine | 11.90211 | 2.30681 | 3.87306 |
| 5.43 | 343.1224 | Maltose | 1.42705 | 0.86045 | 25.69026 |
| 6.38 | 203.1497 | N,N-Dimethylarginine | 0.96167 | 0.82197 | 3.74425 |
| 3.81 | 154.097 | N-γ-Acetylhistamine | 1.20804 | 0.75224 | 3.68103 |
| 2.95 | 166.0858 | Phenylalanine | 1.07319 | 0.71715 | 11.07849 |
| 6.54 | 212.0425 | Phosphocreatine | 0.84048 | 0.33083 | 6.37461 |
| 2.96 | 86.0967 | Piperidine | 1.09633 | 0.86496 | 1.11412 |
| 4.15 | 116.0706 | Proline | 1.50778 | 0.62747 | 7.66805 |
| 2.05 | 218.1381 | Propionylcarnitine | 0.36435 | 0.27349 | 7.47478 |
| 11.33 | 89.1075 | Putrescine | 1.15008 | 0.80505 | 21.42822 |
| 5.87 | 106.0499 | Serine | 1.50221 | 0.90646 | 1.57974 |
| 5.07 | 126.0218 | Taurine | 0.88131 | 1.08258 | 2.31074 |
| 5.23 | 120.0654 | Threonine | 1.28547 | 0.8421 | 3.25291 |
| 4.62 | 150.1121 | Triethanolamine | 1.50258 | 1.11201 | 7.25559 |
| 3.55 | 138.0545 | Trigonelline | 1.92295 | 1.19178 | 6.64802 |
| 3.23 | 205.0965 | Tryptophan | 0.97067 | 0.74245 | 8.60929 |
| 4.44 | 182.0808 | Tyrosine | 1.13226 | 0.76299 | 14.68836 |
| 5.79 | 325.0421 | Uridine 5'-monophosphate | 1.3538 | 0.51604 | 8.25883 |

† Quality Control (QC) S.D. = the Standard Deviation as a percentage of the quantification average metabolite in pooled QC samples across the length of the LC-MS/MS run (see Methods for details).
