# Supplementary material for: Coordinated action of multiple transporters in the acquisition of essential cationic amino acids by the intracellular parasite Toxoplasma gondii
Source: PLoS Pathog. 2021 Aug 25;17(8):e1009835. doi: 10.1371/journal.ppat.1009835 (PMC8423306; doi:10.1371/journal.ppat.1009835)
Supplement: S2 Table — Included are the average retention time (R.T.) of the metabolite, the average mass-to-charge (m/z) ratio of the ion, the name of the metabolite, and the quality control standard deviation (QC Relative S.D). The fold change of the substrate and other detected metabolites was determined by dividing the value at 25hrs with the 0 hr value for TgApiAT1-expressing oocytes, TgApAT6-1 expressing oocytes and uninjected (U.I.) oocytes. (DOCX) [file ppat.1009835.s011.docx]

**S2 Table. Metabolite fold-change upon incubation of *Tg*ApiAT1- or *Tg*ApiAT6-1-expressing oocytes in a solution containing 1 mM Arg for 25 hr.** Included are the average retention time (R.T.) of the metabolite, the average mass-to-charge (m/z) ratio of the ion, the name of the metabolite, and the quality control standard deviation (QC Relative S.D). The fold change of the substrate and other detected metabolites was determined by dividing the value at 25hrs with the 0 hr value for *Tg*ApiAT1-expressing oocytes, *Tg*ApAT6-1 expressing oocytes and uninjected (U.I.) oocytes.

| **Average R.T. (min)** | **Average m/z ion** | **Metabolite name** | ***Tg*ApiAT1**  **Fold change** | ***Tg*ApiAT6-1**  **Fold change** | **U.I.**  **Fold change** | **QC Relative S.D. (%)** |
| --- | --- | --- | --- | --- | --- | --- |
| 2.36 | 204.1225 | Acetylcarnitine | 1.46499 | 1.43142 | 1.4486 | 4.24866 |
| 4.1 | 146.1171 | Acetylcholine | 0.89043 | 1.19091 | 1.37943 | 37.23456 |
| 2.52 | 136.0617 | Adenine | 1.14005 | 0.80371 | 0.79007 | 13.29448 |
| 1.74 | 90.0553 | L-Alanine | 0.94513 | 1.38029 | 1.24116 | 15.04745 |
| 5.35 | 162.0759 | D-2-Aminoadipic acid | 3.67373 | 1.88759 | 6.25677 | 3.33546 |
| 8.58 | 175.1186 | Arginine | 15.28463 | 5.34177 | 2.25544 | 12.30874 |
| 5.64 | 133.0606 | Asparagine | 0.9243 | 1.27216 | 1.21443 | 1.06083 |
| 6.24 | 134.0445 | Aspartic acid | 1.08613 | 1.40711 | 1.33365 | 11.95573 |
| 3.57 | 118.0862 | Betaine | 0.97613 | 1.33291 | 1.21522 | 6.95487 |
| 1.44 | 245.0949 | Biotin | 1.44098 | 1.24744 | 2.00785 | 8.78669 |
| 3.14 | 104.1071 | Choline | 0.97232 | 1.83447 | 1.48795 | 1.21395 |
| 5.89 | 176.1028 | Citrulline | 1.4935 | 0.98924 | 1.49956 | 3.88737 |
| 3.8 | 114.0662 | Creatinine | 0.99703 | 1.12201 | 0.94037 | 7.5295 |
| 4.69 | 122.027 | Cysteine | 0.6494 | 1.05925 | 0.85945 | 11.2624 |
| 5.76 | 148.0601 | L-Glutamic acid | 0.91541 | 1.10707 | 1.16186 | 3.57723 |
| 5.53 | 147.0762 | Glutamine^‡^ | 2.99259 | 4.60584 | 3.63471 | 21.87283 |
| 5.56 | 308.0904 | Glutathione (reduced) | 0.93866 | 1.26923 | 1.13777 | 5.98074 |
| 5.71 | 76.0397 | Glycine | 1.12822 | 2.21901 | 1.21796 | 6.07182 |
| 3.34 | 152.0564 | guanine | 1.28016 | 1.30909 | 1.33412 | 1.77192 |
| 3.33 | 303.1055 | Guanine | 1.42254 | 1.43211 | 1.51345 | 9.52584 |
| 8.59 | 156.0765 | Histidine | 0.92226 | 0.34345 | 1.25509 | 7.04742 |
| 3.45 | 132.1017 | Isoleucine | 0.72937 | 0.89221 | 1.08531 | 2.64271 |
| 3.23 | 132.1013 | Leucine | 0.56509 | 0.50939 | 0.77661 | 7.52253 |
| 8.47 | 147.1125 | Lysine | 0.92825 | 0.09834 | 1.14465 | 9.03476 |
| 3.71 | 150.0582 | Methionine | 0.93695 | 0.21009 | 1.26207 | 8.21433 |
| 3.79 | 126.0661 | 5-Methylcytosine | 1.01437 | 1.11857 | 1.15874 | 7.14846 |
| 8.83 | 133.097 | L-ornithine | 1.18 | 0.12 | 1.28 | 12.6553 |
| 1.74 | 220.1174 | Pantothenic acid | 0.89667 | 1.24022 | 1.13875 | 11.22185 |
| 3.04 | 166.086 | Phenylalanine | 0.87949 | 0.57451 | 1.40975 | 5.25743 |
| 6.55 | 212.0426 | phosphocreatine | 1.33412 | 2.02227 | 1.47017 | 9.38011 |
| 6.98 | 130.0862 | DL-Pipecolinic acid | 1.05231 | 1.1318 | 1.1845 | 24.07875 |
| 4.24 | 116.0706 | Proline | 0.71447 | 0.40158 | 1.17576 | 16.10093 |
| 5.96 | 106.05 | Serine | 1.08253 | 0.97194 | 1.30759 | 3.05111 |
| 5.5 | 343.1226 | Maltose | 1.10792 | 1.49261 | 1.31621 | 9.6986 |
| 5.14 | 126.0219 | Taurine | 0.8664 | 1.21007 | 1.10677 | 7.24399 |
| 5.36 | 120.0656 | Threonine | 0.82325 | 0.71401 | 1.04536 | 3.48497 |
| 3.32 | 205.0968 | Tryptophan | 0.9259 | 1.18321 | 1.15574 | 6.48839 |
| 4.53 | 182.0809 | Tyrosine | 0.97572 | 0.98795 | 1.31038 | 8.01648 |
| 3.52 | 118.0862 | Valine | 0.97613 | 1.33291 | 1.21522 | 6.95484 |

* Fold-change relative to 0 hr incubation time.

‡ Glutamine fold change across conditions incubated with 10mM Arg is replicated in H_2_O-injected oocytes (3.6-fold), indicating this increase occurs independently of cRNA injection and protein expression.
