# Supplementary material for: Coordinated action of multiple transporters in the acquisition of essential cationic amino acids by the intracellular parasite Toxoplasma gondii
Source: PLoS Pathog. 2021 Aug 25;17(8):e1009835. doi: 10.1371/journal.ppat.1009835 (PMC8423306; doi:10.1371/journal.ppat.1009835)
Supplement: S3 Table — (DOCX) [file ppat.1009835.s012.docx]

**S3 Table.** **Solution composition used for uptake and electrophysiological recordings** **in *X. laevis* oocytes**

| **Buffer** | **[Na^+^] (96 mM)** | **[K^+^] ( 2 mM)** | **[Cl^-^] (96 mM)** | **[Mg^2+^] (1 mM)** | **[Ca^2+^] (1.8 mM)** | **Buffer** | **Replaced 🡪 Replacement salt** | **Titration (pH)** |
| --- | --- | --- | --- | --- | --- | --- | --- | --- |
| **Salt Adjustable Buffers** (electrophysiology and uptake) | | | | | | | | |
| ND96 | NaCl | KCl | NaCl | MgCl_2_ | CaCl_2_ | HEPES 5 mM | $-$ | NaOH (7.3) |
| KD96 | $-$ | KCl | KCl | MgCl_2_ | CaCl_2_ | HEPES 5 mM | Na^+^$\to$ K^+^ | NaOH (7.3) |
| Gluconate96 | KC_6_H_11_O_7_ | NaC_6_H_11_O_7_ | $-$ | Mg(C_6_H_11_O_7_)_2_ | Ca(OH)_2_ | $-$ | $\mathrm{Cl}^{-}\to$ $C_{6}H_{11}O_{7}^{-}$ | HCl (7.3) |
| ChCl96 | C_5_H_14_NOCl | KCl | C_5_H_14_NOCl | MgCl_2_ | CaCl_2_ | HEPES 5 mM | Na^+^$\to C_{5}H_{14}NO^{+}$ | NaOH (7.3) |
| ND96 ($-$CaCl_2_) | NaCl | KCl | NaCl | MgCl_2_ | $-$ | HEPES 5 mM | $\mathrm{Ca}^{2+}\to\mathrm{Mg}^{2+}$ | NaOH (7.3) |
| **Salt Adjustable Buffers** (uptake experiments only) | | | | | | | | |
| NMDG96 | NMDG^*^-Cl | KCl | NMDG-Cl | MgCl_2_ | CaCl_2_ | HEPES 5 mM | Na^+^$\to$ NMDG | HCl (7.3)^†^ |
| ND96 ($-$K^+^) | NaCl | $-$ | NaCl | MgCl_2_ | CaCl_2_ | HEPES 5 mM | $K^{+}\to\mathrm{Mg}^{2+}$ | NaOH (7.3) |
| ND96 ($-$Mg^2+^) | NaCl | KCl | NaCl | $-$ | CaCl_2_ | HEPES 5 mM | $\mathrm{Mg}^{2+}\to K^{+}$ | NaOH (7.3) |

^*^NMDG (N-methyl-D-glucamine)

^†^Chloride is added to the buffer as HCl during the neutralisation of the free base form of NMDG
