# Supplementary material for: Coordinated action of multiple transporters in the acquisition of essential cationic amino acids by the intracellular parasite Toxoplasma gondii
Source: PLoS Pathog. 2021 Aug 25;17(8):e1009835. doi: 10.1371/journal.ppat.1009835 (PMC8423306; doi:10.1371/journal.ppat.1009835)
Supplement: Attachment — Submitted filename: Response to reviewers.pdf [file ppat.1009835.s013.pdf]

Response to Reviewers

Reviewer queries and comments are in black, and our responses to them in blue. All changes to the text of the manuscript are highlighted in red in the marked file. In addition to addressing the reviewer comments, we have made additional changes to the manuscript to address typographical errors, and to improve the clarity of the descriptions of the experiments that we performed. These changes are also highlighted in red text on the marked file.

Part II – Major Issues: Key Experiments Required for Acceptance

Reviewer #2

1. The proposed function of TgApiAT6-1 requires that it is found in the Toxoplasma membrane. While the localized is based on homology, this approach does not provide the necessary clearly evidence that TgApiAT6-1 localizes in the parasite membrane. It needs to be established that TgApiAT6-1 is indeed the membrane using a technique such as immunofluorescence using intra and extracellular parasites.

We have demonstrated in a previous manuscript that *Tg*ApiAT6-1 localises to the periphery of the parasite, overlapping with the plasma membrane marker *Tg*P30 (PMID 30742695). We have added the following text to the manuscript to make this clearer to the reader (changed text underlined): “In a previous study of the ApiAT family in *T. gondii*, <u>we demonstrated that *Tg*ApiAT6-1 localized to the plasma membrane of the parasite [ref].</u>” (Lines 129-130)

2. Given that TgApiAT6-1 depletion results in defects on arginine/lysine uptake and severe replication defect may mostly be related to the change in availability of intracellular amino acids. However, the current study does not establish the replication of rTgApiAT6-1 in presence of different concentrations of arginine and/or lysine. A more thorough investigation of parasite replication in TgApiAT6-1-depleted parasites, including a doubling assay/proliferation assay in RPMI and DMEM, may help establish why TgApiAT6-1 is essential.

We agree and have conducted two proliferation assays with *Tg*ApiAT6-1 parasites cultured in the absence or presence of ATc, one in which the concentration of Lys was varied in the presence of a constant Arg concentration, and another in which the concentration of Arg was varied in the presence of a constant Lys concentration (S6 Fig). We found that regardless of the concentration of Lys or Arg in the growth medium, parasites in which *Tg*ApiAT6-1 was depleted were unable to proliferate. This indicates that, unlike *Tg*ApiAT1 (PMID 28205520), proliferation defects in parasites lacking *Tg*ApiAT6-1 cannot be rescued by altering the concentrations of the substrates of this transporter. Together with our other data, these experiments support the hypothesis that *Tg*ApiAT6-1 performs an essential role in Lys uptake by the parasite. We have described the data and these conclusions in the text (Lines 266-277; Lines 298-305)

3. In the proposed working model (figure 7), the authors speculate that in cells with low arginine concentration (e.g liver) TgApiAT6-1 would preferentially uptake lysine intend of arginine, while in cells with high arginine concentration (e.g. kidneys), arginine and lysine are uptake by TgApiAT6-1. If this is the case, the authors should show the parasite replication in kidneys cell line (HEK 293) and liver cell line (HepG2) in presence or absence of arginine and/or lysine to help understand the role of TgApiAT6-1 in acute infection. Also, it will help to understand

the role of TgApiAT6-1 by infection of the animal model with WT, rTgApiAT6-1 and cTgApiAT6-1 parasite and analyze the animal mortality.

The reviewer proposes some interesting experiments. In a related study, we have developed a luciferase-based assay that allows us to estimate the arginine that parasites encounter in their host cells (both in vitro and in vivo; manuscript available on BioRxiv (https://www.biorxiv.org/content/10.1101/798967v1). We demonstrate in that study that parasites likely encounter higher concentrations of Arg in the kidneys than in the liver. We also show in that study that knockdown of *Tg*ApiAT6-1 leads to a depletion of [Arg] in the parasite, and a compensatory increase in the abundance of the *Tg*ApAT1 arginine transporter. We incorporate these observations from the related study in the model we present in Figure 7.

Regarding the experiments that the reviewer proposes: the concentration of [Arg] and [Lys] that parasites encounter in *in vitro* cell culture systems will largely reflect the Arg and Lys present in the culture medium. Given that this is a very artificial system compared to what parasites will encounter in a host organism, we will likely observe minimal differences in using different host cell types. We believe that the *in vivo* evidence from our related manuscript provides the strongest support for the model we present in Figure 7.

Regarding the animal infection experiments to test the importance of *Tg*ApiAT6-1 for parasite virulence, we have previously tested parasite virulence in a mouse infection with strains of parasites wherein genes-of-interest had been knocked out. We have tried to knockout *Tg*ApiAT6-1 using a CRISPR/Cas9 genome editing strategy without success (despite demonstrating that we could target the *Tg*ApiAT6-1 locus with the sgRNAs that we were using; see the description of these experiments in PMID 30742695). We could test virulence using the ATc-regulatable *Tg*ApiAT6-1 strain in a mouse model by infecting mice and adding ATc to the drinking water, however our lab has not previously undertaken mouse infection experiments using ATc-regulatable mutants. Given the evidence we provide that both *Tg*ApiAT6-1 and Lys are important for parasite proliferation in a tissue culture system (Fig 1B-D, S6 Fig and S7 Fig), we have instead added a caveat to our interpretation that *Tg*ApIAT6-1 is essential (change underlined): "Our study establishes that *Tg*ApiAT6-1 is essential for tachyzoite proliferation *in vitro*, most likely due to its role in uptake of the essential amino acid Lys." (Lines 429-430)

Part III – Minor Issues: Editorial and Data Presentation Modifications

Reviewer #1

Abstract: the authors should indicate that their experimental model was both with T. gondii parasites as well as transport studies in Xenopus laevis oocytes.

We have modified the text to read (change underlined): "Using a combination of parasite assays and heterologous characterisation of *Tg*ApiAT6-1 in *Xenopus laevis* oocytes, we demonstrate that *Tg*ApiAT6-1 is a general cationic amino acid transporter that mediates both the high-affinity uptake of lysine and the low-affinity uptake of arginine." (Lines 26-27)

Figure 1: Panel A should include units for molecular weights (i.e. kDa). We have added this to the figure. What concentration of aTC was used? We state in the methods that "Where applicable, anhydrotetracycline (ATc) was added to a final concentration of 0.5 μg/ml with 0.025 % (v/v) ethanol added to relevant vehicle controls." (Lines 545-547). Were different concentrations tested (i.e. titration)? Dose-response data, if available, should be included in the supplement. We did not titrate the ATc concentration. We demonstrate that the concentration of

ATc that we use in our experiments is not deleterious to proliferation of WT parasites (Fig 1C). From past experience, increasing the concentration of ATc beyond 0.5 μg/ml can lead to effects in parasites unrelated to knockdown of the target gene.

Figure S1: Please explain in the text how this regulatable system works, in terms of the role of the teto-sag4 and how repression is achieved.

We have added the following text to the S1 Fig legend, and additionally cite the Meissner et al 2002 paper where this system was established in *T. gondii*: "The teto7-sag4 promoter is bound by a tetracycline-controlled transactivator protein that facilitates transcription of the downstream gene (*Tg*ApiAT6-1 in this instance). The addition of the tetracycline analogue ATc to the culture medium results in binding of ATc to the transactivator protein, which inhibits binding of the transactivator protein to the teto7-sag4 promoter, and consequently reduces transcription of the downstream gene [ref]" (Lines 1246-1251)

Figure 2: When describing the parasite amino acid data in panel A, the authors should add a note that Arg was not detected by GC-MS (it would help to also explain why).

This is a known limitation of GC-MS. Sample derivatisation typically involves trimethylsilylation, which converts Arg to ornithine (PMID: 15618298). We have modified the text to reflect this (relevant change underlined): "These data suggested that *Tg*ApiAT6-1 may be a Lys transporter, although it could also mediate the uptake of other amino acids not detected under the transport conditions of the experiments, or not detected by GC-MS, such as Arg. Arg is readily converted into L-ornithine during in-line heating and separation for GC-MS such that very little remains chemically unmodified [refs]. " (Lines 160-164)

In Panels B-D, the background uninfected oocyte should be shown or listed somewhere in a supplemental table. There is no label on the first bar of Panel D. The x-axis label on Panel F should be [L-Arginine]. The labels on the axes are very small and hard to read.

We have incorporated the reviewer's suggestions. Specifically, we have added the uninjected oocyte background uptake for all substrates shown as a new supplementary figure (S3 Fig). S3A Fig shows the uninjected background uptake that was used to determine the *Tg*ApiAT6-1-mediated uptake shown in Fig 2B. Similarly, S3B-C Fig show the uninjected background for Fig 2C-D. S3D-E Fig show the Lys and Arg uptake in H_2O-injected control oocytes that were subtracted from the data in Fig 5E-F. We have changed amino acid labels in all figures to single letter codes, and provided shorter abbreviations for amino acid metabolites i.e. Pu (Putrescine), Sp (Spermidine) and GA (GABA), which are listed in the appropriate figure legends. The X-axis label for Fig 2F has also been adjusted (to "[Arg]" to be consistent with other axis titles in the manuscript – see our response to this reviewers comment on Figure 3). Text has been added to the figure legends to describe the additional figures.

How did the authors select the concentrations of amino acids used in these assays?

The concentration of the substrates we tested in the uptake assays (100 μM; Fig 2B) was in the ball-park of the $K_{0.5}$ values that we had determined for the substrates of other ApiAT transporters (PMIDs 28205520 and 30742695), and within the range of reported intracellular concentrations of the amino acid in mammalian host cells. For the competition assays (Fig 2C-D), we used the competing substrates at 10X and 100X the concentration of the substrate we were measuring uptake for. We state in the manuscript that "we measured *Tg*ApiAT6-1-mediated uptake of Arg in oocytes in the presence of a 10-fold (Fig 2C) or 100-fold (Fig 2D) higher

concentration of other, unlabelled amino acids" (Lines 181-183).

It would be interesting to show the predicted topology of this transporter. Homology with other ApiAT proteins should be shown in the supplement. Based on the topology of major facilitator superfamily (MFS) transporters where this has been experimentally determined (e.g. PMIDs 11932679 and 22458847 and references therein) it is likely that *Tg*ApiAT6-1 has 12 transmembrane domains, with the N- and C-termini, and the loop between transmembrane domains 6 and 7, in the cytosol. Based on protein alignments, we predicted in a previous study that ApiAT family proteins have 12 transmembrane domains (S1 Fig in PMID 30742695), We have previously presented a detailed protein alignment and phylogenetic analysis of *Tg*ApiAT6-1 and other ApiAT proteins (S1 Fig and Fig 1 in PMID 30742695), and we summarise this study in the introduction of the present manuscript.

Table 1: Vmax levels should be shown. Also, please clarify the meaning of K0.5 in the footnote. We have included Vmax values in Table 1. We have added the following to the footnote text in Table 1 to describe $K_{0.5}$: "*Michaelis constant, the substrate concentration at half the maximal velocity of the transporter". (Line 1097)

Figure 3: In Panels A-C, the short horizontal lines indicating the drug pulse are not clear – maybe arrows or vertical lines should be added? The horizontal lines indicating the arginine pulses have been changed to dashed lines to make them clearer. The usage of the dashed lines has also been indicated in the figure legend. In Panel E, Arginine is spelled out when it is abbreviated everywhere else. The use of 'Arg' and 'Lys' has been made consistent throughout the figures. For Panels E-F, the length and timing of Arg pulse was not indicated. All current measurements were steady-state, reflecting the move to a new constant current measurement following the continued application of substrates. Typically, as can be observed in the tracing for Fig 3A, this occurred after 5-10 min of continual application. We have added the following text to the figure legend to report this information (change underlined): "All oocytes were voltage-clamped at −50 mV and exposed to a range of Arg concentrations and at of one of 3 concentrations of Lys (0, 50, 500 μM) until a new steady-state current baseline was achieved". (Lines 1161-1164). Also, the error bars in panel E are large. How can the authors be confident that the Michaelis-Menten fit is correct? We report the R^2 values for goodness of fit of the model to the data in the figure legend that accompanies Figure 3E and 3F. These suggest a good fit for the 0 μM and 50 μM Lys conditions ($R^2 \geq 0.84$), and less confidence in the fit for the 500 μM condition ($R^2 \sim 0.65$).

Figure 4: It would be interesting to do Lys and Arg competition assays in the knockdown parasite, varying one amino acid concentration to test its ability to inhibit transport of the other radiolabeled amino acid (at a fixed concentration) to determine the relative contribution of this transporter to Lys and Arg transport. This is not essential and have technical caveats but it would be useful to add if the data are available. We agree that it would be interesting to undertake a detailed analysis of this, but we have not done so here. We have demonstrated in a previous study that uptake of Lys by the parasite (which we demonstrate in this manuscript is mediated by ApiAT6-1) is inhibited by Arg as well as the cationic amino acid ornithine (Figure 5b in PMID 28205520), and that Arg uptake in parasites lacking *Tg*ApiAT1 is inhibited by Lys (Supplementary Figure 7a in PMID 28205520) . We have added the following text to note this: "We demonstrated in a previous study that Lys uptake by the parasite is inhibited by Arg, and that Arg uptake in parasites lacking the selective Arg transporter *Tg*ApiAT1 is inhibited by Lys [ref]. This is consistent with the competition between these substrates for uptake by *Tg*ApiAT6-1 that we observed in the oocyte experiments (Fig 3E-F)." (Lines 248-252)

Figure S4: Transport does not appear to plateau at 20 min, especially with Arg. It would have been preferable to conduct these studies over a longer time frame. We did not extend these assays beyond 20 min.

Figure S5: A diagram of the TgLysA locus and the location of the catalytic domain would be useful. We have included a diagram of the *Tg*LysA protein drawn to scale, with the site of the frameshift mutation and the positions of the predicted active site residues of the protein depicted (S7A Fig). We have modified the text of the figure legend to describe this diagram. The authors mention that proliferation is inhibited below 50 uM, but there are so many points on the graph below 200 uM [Lys] that it is difficult to see this on the graph. Please add more minor ticks. We have redrawn this figure so that the x axis is on a log_{10} scale. This makes the positions of the data points clearer (now S7B Fig).

Figure 5: It is unclear why the experiment done in Panel A with TgApiAT6-1 and Lys was not also done with Arg – can the authors clarify. The purpose of the experiment in Fig 5A was to establish whether transport of substrates by *Tg*ApiAT6-1 could be *trans*-stimulated by substrates on the other side of the membrane. We tested this for Lys, since it was a validated substrate. Having demonstrated that transport of Lys by *Tg*ApiAT6-1 could be *trans*-stimulated, we went on to show that transport of Arg by *Tg*ApiAT6-1 could also be *trans*-stimulated by a range of substrates, including Arg (Fig 5C). For Panels C-D, the Y-axis should be labeled Arg efflux to clarify. These data are not only showing Arg efflux (Arg_o), but also, as indicated by the figure keys, Arg retention in oocytes (Arg_i). Labelling the X-axis 'Arg' with a rate unit per oocyte is the appropriate title. Experiments done in Panel C with TgApiAT6-1 should also preferably be done with Lys. We have performed the requested Lys efflux/retention experiment in the presence of various candidate *trans*-substrates, and include these data as S3F Fig. These data reveal that specificity of the tested candidate *trans*-stimulating substrates in Lys efflux is similar to that observed with candidate *trans*-stimulating substrates on Arg efflux (Fig 5C). Panel F is similar to Panel 2C-D – this should be spelled out and experimental differences between those data stated. We specify in the figure legend that the uptake experiments in Fig 5E-F were performed with "oocytes pre-loaded with a range of candidate *trans*-stimulating substrates." Fig 2C-D are measuring Arg uptake in competition with unlabelled amino acids on the same (*cis*) side of the membrane, and we specify this in the figure legend by stating that we are measuring "Inhibition of Arg uptake into *Tg*ApiAT6-1-expressing oocytes by a range of amino acids."

Figure 6: 50-54 hours was much longer than the other experiments. Why use this time course in this experiment and not others? The purpose of the LC-MS/MS experiments in oocytes was to establish whether *Tg*ApiAT1 and *Tg*ApiAT6-1 were capable of net uptake of their respective substrates, as opposed to obligatory one-for-one exchange of substrates as looked possible in the efflux experiments conducted in Figs 5A and 5B. Oocytes are very large cells with a volume of 380-500 nl/oocyte for the mature stage oocytes we used in our experiments. Hence, they require some time to accumulate substrate to the levels required to test whether net substrate uptake is occurring. We have reworded the description of these experiments in the results section to make this clearer (changes underlined): "*Tg*ApiAT6-1 or *Tg*ApiAT1-expressing oocytes were incubated in a solution containing 1 mM Lys or Arg for 50-54 hr, a timeframe that enabled us to observe the accumulation of specific amino acids over time, which we then quantified using external calibration curves (S8A-B Fig)." (Lines 389-391). The LC-MS/MS technique that we used is highly advantageous for this type of analysis since, unlike radiolabelled uptake, it allows for actual concentration changes to be observed from membrane flux in real time, while simultaneously accounting for any metabolic conversion of the fluxed substrates (Lys and Arg in this case). Also, in the parasite both Arg transporters are indicated to be present on the parasite plasma membrane. Have the

authors examined relative expression levels of these two proteins in the parasite? We've never directly compared abundances of *Tg*ApiAT6-1 and *Tg*ApiAT1, but we demonstrate in another manuscript (available on BioRxiv; https://www.biorxiv.org/content/10.1101/798967v1.full) that *Tg*ApiAT6-1 is constitutively expressed in the parasite at a range of [Arg] in the culture medium, whereas the abundance of *Tg*ApiAT1 varies with [Arg]. So the relative expression levels of these proteins depends on Arg availability. We discuss the implications of this in the context of the model we present in Figure 7: "The differential expression of *Tg*ApiAT1 may therefore allow these parasites to survive when Arg levels are limited, while *Tg*ApiAT6-1 may ensure regulated uptake of Arg and Lys under nutrient rich conditions." (Lines 437-439)

Figure 7: This model fits with the conclusions of the paper. Is the Em of oocytes tested in this study consistent with that of parasites? It would be beneficial to test whether a membrane potential is important for transport. We note in the manuscript the following (with minor changes underlined): "Extracellular *T. gondii* tachyzoites are known to have an inwardly negative E_m [ref]. Whether the same is true of intracellular tachyzoites, the stage at which parasite proliferation is dependent on Arg and Lys uptake, has not been determined." (Lines 502-504.) A challenge for the future will be to establish approaches to determine the Em of the intracellular stage of the parasite – at present we have no good way to do this.

Points to consider for the discussion:
Could these transporters be a drug target? It would be interesting to explore this concept and discuss whether selective inhibitors could be screened for. These are interesting ideas. Given their importance for parasite proliferation (and in the case of *Tg*ApiAT1, parasite virulence), inhibitors of these transporters could be effective drugs. We have not undertaken any drug screening studies, but one could imagine a screen to identify inhibitors that target *Tg*ApiAT1 by testing for inhibitors of proliferation when parasites are cultured in low Arg (conditions in which *Tg*ApiAT1 is required for parasite proliferation) compared to when they are cultured in high Arg (conditions in which *Tg*ApiAT1 is dispensable for parasite proliferation). Given that these ideas are still speculative, we've chosen not to address this point in the manuscript. Also, do these ApiAT proteins have any naturally occurring mutations? The amino acid sequences of both *Tg*ApiAT6-1 or *Tg*ApiAT1 are highly similar in the 15 strains of *T. gondii* for which sequence data are available on www.ToxoDB.org. There are a few differences in the amino acid sequences of *Tg*ApiAT1 and *Tg*ApiAT6-1 between different strains of *T. gondii*, although the role(s) of these residues in protein function are not known. While we are very interested in future to map things like substrate binding sites of *Tg*ApiAT1 and *Tg*ApiAT6-1, we believe that commenting on this in the manuscript is speculative and have chosen not to address this point.

Typographical comments:

Pg. 13: Arg written as Arg+. Changed to "Arg"
Pg. 16: "twofold" to "two-fold"; keep consistency with writing out numbers or not. Changed to "two-fold" as suggested. Elsewhere, we have now followed the convention of writing out numbers less than 10 (so "five-fold" instead of "5-fold", unless decimal places are included *e.g.* "4.7-fold") and using numerals for numbers 10 and above (so "16-fold" instead of "sixteen-fold")
Pg. 17: "three-fold" See previous comment
Figure 5: In the figure legend, "IUPAC 3-latter codes" should be "IUPAC 3-letter codes. We have replaced the 3-letter with the 1-letter amino acid codes in Fig 2 and 5 in response to other reviewer comments.

References are missing italics. Corrected

Figure 6: The legend has a typo: “A. A-C.” Corrected

Reviewer #2

Fig. 1B: Absence of TgApiAT6-1 decreases parasite proliferation, however after 3-4 days of ATc treatment the parasites are still viable? Can rTgApiAT6-1+ATc infect new cells? We have not tested invasion competency of r*Tg*ApiAT6-1 parasites cultured in the presence of ATc. We show that parasites are able to uptake/metabolise the glucose analog 2-deoxyglucose after 2 days culturing in ATc (S5E Fig). We state in the manuscript that “uptake of 2-deoxy-glucose, a glucose analogue, was unaffected upon *Tg*ApiAT6-1 knockdown (S5E Fig). These data indicate that the observed defects in Lys and Arg uptake in the r*Tg*ApiAT6-1 strain were not the result … of a general impairment of solute uptake or parasite viability.” (Lines 262-265)

Fig. 1D: What is the expression level of cTgApiAT6-1? Is it similar to WT levels? Given that cTgApiAT6-1 replicates faster than WT according to Fig. 1D. We have not been able to test the relative expression levels of the WT ApiAT6-1 vs the complemented *Tg*ApiAT6-1 proteins, since these differ in the epitope tags that we have added to them (1xHA for the WT ApiAT6-1 reported in PMID 30742695, and 3xHA for the complemented ApiAT6-1 reported in this manuscript). The fluorescence proliferation assays depicted in Fig 1D are sensitive to the viability of the parasites that we use in the initial inoculations. If one parasite strain used in an assay is less viable than another strain (which might occur if, for example, that strain egressed from host cells a few hours sooner than the other strain), fewer parasites of the less viable strain will invade host cells following inoculation, leading to an apparent reduction in replication rate in that strain for that particular experiment. We are therefore cautious about comparing data between strains for these sorts of experiments. The key comparison is the difference in proliferation between parasites from a single strain (which derive from the same population of parasites, and therefore have the same initial viability) cultured in the absence or presence of ATc. So we observe no difference in proliferation between WT parasites cultured in the absence or presence of ATc, or between rTgApiAT6-1/cTgApiAT6-1 cultured in the absence or presence of ATc, but a large difference between rTgApiAT6-1 parasites cultured in the absence or presence of ATc. We have reworded this section of the manuscript, since the original text did compare growth rates of WT and rTgApiAT6-1 parasites cultured in the presence of ATc (changes underlined): “<u>Compared to parasites cultured in</u> the absence of ATc<u>, we observed a major defect in proliferation in</u> r*Tg*ApiAT6-1 <u>parasites cultured in the presence of ATc</u> (conditions under which *Tg*ApiAT6-1 is knocked down<u>;</u> Fig 1B). ATc had no effect on the proliferation of wild type (WT) parasites under the same conditions (Fig 1C).” (Lines 140-144). As we note in the manuscript, we conclude from these experiments that *Tg*ApiAT6-1 is important for parasite proliferation.

Given that TgApiAT1 is upregulated in arginine limitation. What is the expression TgApiAT6-1 levels in parasite cultivate in lys/arg limitation? We report in a separate manuscript (https://www.biorxiv.org/content/10.1101/798967v1.full) that *Tg*ApiAT6-1 expression does not change with changing [Arg] in the culture medium.

Reviewer #3

1. Lines 152 to 155. Was there a rationale for using 2 mg/ml labelled amino acids and a 15 min incubation time? The experimental conditions were adopted from a previous study looking at ^{13}C amino acid uptake in extracellular parasites (PMID: 30742695). We state in the manuscript that "Labelling experiments with a mixture of [^{13}C]-amino acids were performed as described previously [ref]." (Lines 626-627). The concentration of ^{13}C-amino acids is similar to RPMI medium, which contains about 1mg/ml of amino acids.

2. Line 158-159. The authors state other amino acids (other than lysine) could be transported by TgApiAT6-1 that are not detected by GC-MS. Another explanation is that the conditions used do not allow the detection of transport of other amino acids (this is covered 2 paragraphs later but should at least be noted here). We have modified the text to read (change underlined): "These data suggested that *Tg*ApiAT6-1 may be a Lys transporter, although it could also mediate the uptake of other amino acids <u>not detected under the transport conditions of the experiment, o</u>r not detected by GC-MS, such as Arg." (Lines 160-163)

3. Line 257-276. The authors genetically disrupt TgLysA which has no effect on parasite proliferation over a range of lysine concentrations compared with wild type parasites. While I agree that the disruption would most likely render the truncated enzyme non-functional, there is no proof (phenotypic or functional) that this is the case, as the enzyme would seem to be non-essential. Perhaps, at the very least, the authors could note that this is consistent with previous CRISPR studies (ref [33]?), if that is the case (apologies if I missed this in the text somewhere). We agree. We have modified the text to read (change underlined): "The resultant 2 bp deletion in the *Tg*LysA locus causes a frameshift mutation in the *Tg*LysA open reading frame, leading to the <u>predicted</u> production of a truncated *Tg*LysA protein lacking residues 281 to 686, and a loss of key active site residues that are predicted to render the enzyme non-functional". (Lines 285-288). We have also added the following sentence to the end of this paragraph: "<u>The observed dispensability of *Tg*LysA for tachyzoite proliferation is consistent with data from an earlier CRISPR-based screen that examined the importance of all genes encoded in the parasite genome [ref]</u>." (Lines 294-296)

4. Discussion/Figure 7. The authors provide a model for cationic amino acid uptake into T gondii-infected cells, using the kidney and the liver as examples of differing cationic amino acid environments. In the model, host CAT1 is noted (based on a study reporting infection induced upregulation of CAT1 but not CAT2 or 3 in a range of cells). However, I'm not sure liver cells were used in that study. Liver cells express CAT2A/B as the predominant cationic amino acid transporters. These have been shown to be important for liver stage malarial parasite development (Meireles et al 2017; PMID: 28642498). Perhaps this could be noted. We thank the reviewer for these insights and suggestions. The Augusto study we reference (PMID 31194856) observed CAT1 upregulation upon *T. gondii* infection in mouse embryonic fibroblasts and no major changes in CAT2 or CAT3 mRNA levels in these host cells (although the latter observations may be a consequence of the cell type in which this was measured). To address the reviewer's points, we have modified the text to note that increased Lys uptake into host cells is likely to occur in host cells where CAT1 is the major AA^+ transporter (change underlined): "Upregulation of CAT1 is therefore likely also to cause an increase in Lys uptake in host cell<u>s where CAT1 serves as the major AA^+ transporter</u>." (Lines 444-446). We have also placed a similar caveat on a conclusion we make later in the discussion (changed underlined): "This increases AA^+ uptake into host cells <u>in which CAT1 is the major AA^+ transporter</u>, while maintaining the Arg:Lys ratio from the extracellular milieu." (Lines 461-463). Additionally, we have added some text to note the uncertainty around what happens in host cells where other AA+ transporters (like CAT2) have a major role in AA^+ uptake, and a sentence noting the Meireles study in *P.*

berghei and how that may be relevant to *T. gondii*: "It remains to be seen whether Lys and Arg uptake into host cells increases in response to parasite infection in host organs in which other host AA^+ transporters contribute to uptake (e.g. in the liver, where CAT2 has a major role in facilitating AA^+ uptake; [ref]). In this context, it is notable that liver stage development of P. berghei parasites is impaired in mice in which CAT2 has been knocked out [ref], and it is conceivable that the roles of TgApiAT6-1 and TgApiAT1 in Arg uptake may differ in host cells that express different AA+ transporters." (Lines 451-457). Finally, we have modified Figure 7 to include a "?" next to the CAT2 transporter in the "liver" section of the diagram to note the uncertainty around whether host AA+ uptake increases in tissues where CAT2 has a major role in AA+ uptake. We have also modified Fig 7 to make clearer the roles of Arg synthesis and catabolism in the model.

5. Methods. Please note the specific activity of the radiolabels used. We have added the specific activities of each radiolabelled substrate to the Methods section. (Lines 714-719)

6. Line 670/S2 Fig. A. For the oocyte protein detection assays, was each well loaded with equal amounts of protein and how much? We controlled the number of oocytes used per condition. We now specify in this in the methods text (change underlined): "Oocyte surface biotinylation and whole membrane preparations were performed as described previously [refs], using equivalent numbers of oocytes per experiment." (Lines 726-727). We also note this in the S2 Fig legend: "Each lane contains protein equivalents from equal oocyte numbers." (Line 1259)

7. Figures in general. I reviewed the manuscript printed on A4 paper and was unable to read many of the labels (particularly the amino acid abbreviations). As outlined in our response to a similar comment from Reviewer 1, we have modified all 3-letter IUPAC amino acid codes to 1-letter codes and use shorter abbreviations for amino acids and amino acid metabolites. We have also enlarge the axis and key text for most small text figures and enlarged figures in general.

8. Figure 5 legend and possibly other places. The authors note in several places that "microinjecting 5 mM of Arg". Perhaps this could be rephrased to "microinjecting Arg to a concentration of 5 mM" as it reads currently as though you are injecting a solution of 5 mM of Arg. We agree, and have changed this in the Figure 5 legend as suggested. This now reads (changes underlined): "Oocytes were pre-loaded (PL) with Arg by first microinjecting Arg to a final concentration of ~5 mM … *Tg*ApiAT6-1-injected oocytes were pre-loaded by microinjecting the indicated substrates to a final concentration of ~5 mM …" (Lines 1186-1187, Lines 1201-1202). We went through the manuscript and couldn't find any other instances where this wasn't clear. We already stated in the main body of the text that: "Oocytes expressing *Tg*ApiAT6-1 were microinjected with a range of amino acids and arginine-derived metabolites to a final concentration of approximately 5 mM…" (Lines 355-357)

9. Figure S2 legend. For the description of unlabelled and labelled Arg and Lys 100 microM seems to mentioned too many times. Removed second "100 μM Lys/Arg" in each of these sentences as suggested.

10. General. The authors often state that transport values in negative controls have been subtracted from the data presented in the figures. If possible, please provide an estimate of these transport measurements so as to give an idea of signal to noise. We have added a new figure (S3 Fig) to report uptake data for uninjected or H_2O-injected control oocytes not expressing *Tg*ApiAT6-1 or *Tg*ApiAT1. S3A-C Fig report the background for Fig 2B-D,

while S3D-E Fig report the background for the *trans*-stimulated uptake experiments in Fig 5E-F. We have added notes in the figure legend text referring to these data, and relabelled all later supplementary figures to reflect the new numbering.

11. Typos ("" add/[] remove): Line 49, concentrations “are” comparatively. Changed as suggested. Line 52, able “to” survive. Changed as suggested. Line 108, important for [the] tachyzoite. Changed as suggested. Fig 3 legend, line 11, and at [of] one. Changed as suggested.
